# Aloine, the Cathartic Principle of Aloes

**Published:** 1851-09

**Authors:** 


*Aloine, the Cathartic principle of Aloes.*—Messrs. T. & H. Smith, report in the Monthly Journal of Medical Science, Feb. 1851, the discovery of a new principle in Aloes, on which the cathartic properties of the drug appear to depend. *Aloine* is a neutral crystalline substance, of a deep yellow color, soluble in very small quantity in cold water, readily soluble in hot water, alcohol, alkaline liquids, acetic ether, and acetic acid. It produces the cathartic action of aloes in doses of from one to two grains, and there can be little doubt of its being the active principle.

